# Integrated immunogenomic analysis of single-cell and bulk tissue transcriptome profiling unravels a macrophage activation paradigm associated with immunologically and clinically distinct behaviors in ovarian cancer

**DOI:** 10.1016/j.jare.2022.04.006

**Published:** 2022-04-15

**Authors:** Congcong Yan, Ke Li, Fanling Meng, Lu Chen, Jingting Zhao, Zicheng Zhang, Dandan Xu, Jie Sun, Meng Zhou

**Affiliations:** a School of Biomedical Engineering, Wenzhou Medical University, Wenzhou 325027, P. R. China; b Department of Gynecology, Harbin Medical University Cancer Hospital, Harbin 150081, P. R. China; c School of Basic Medical Sciences, Harbin Medical University, Harbin 150081, P. R. China

**Keywords:** Macrophages, Single-cell, Artificial neural network, Immune microenvironment

## Abstract

**Introduction:**

Increasing evidence demonstrates that the activation states and diverse spectrum of macrophage subtypes display dynamic heterogeneity in the tumor microenvironment, which plays a critical role in a variety of cancer types.

**Objectives:**

To investigate the heterogeneity and the homeostasis of different macrophage subtypes, as well as their effect on biological and clinical manifestations of ovarian cancer (OV).

**Method:**

Integrated immunogenomic analysis of single-cell and bulk tissue transcriptome profiling was performed to systematically investigate the association between macrophage activation and prognostic and therapeutic efficacy. Consensus clustering analysis was used to define novel macrophage subtypes. An artificial neural network was used to simulate the dynamic activation of macrophages.

**Results:**

The pan-cohort results suggested that high relative infiltration abundance of M0 and M1 macrophages was associated with improved outcome and therapeutic efficacy. However, it was the opposite for M2 macrophages. Unsupervised consensus clustering analysis revealed two OV subgroups characterized by a balance between M0, M1 and M2 macrophages with distinct clinical and immunological behaviors. Finally, a macrophage polarization-derived artificial neural network model was proposed to serve as a robust prognostic factor and predictive biomarker for therapeutic efficacy, which was validated in different independent patient cohorts.

**Conclusion:**

The present study provides a new understanding of macrophage heterogeneity and its association with OV prognosis and underlines the future clinical potential of a macrophage activation model for tumor prevention and treatment.

## Highlights

• Integrated immunogenomic analysis of single-cell and bulk tissue transcriptome profiling was conducted.
• Two novel OV subgroups were characterized by a balance between M0, M1 and M2 macrophages with distinct clinical and immunological behaviors.
• A macrophage polarization-derived artificial neural network model (MacroANN) was developed to predict patient outcomes and therapeutic efficacy.

## Graphical abstract

**Figure:**
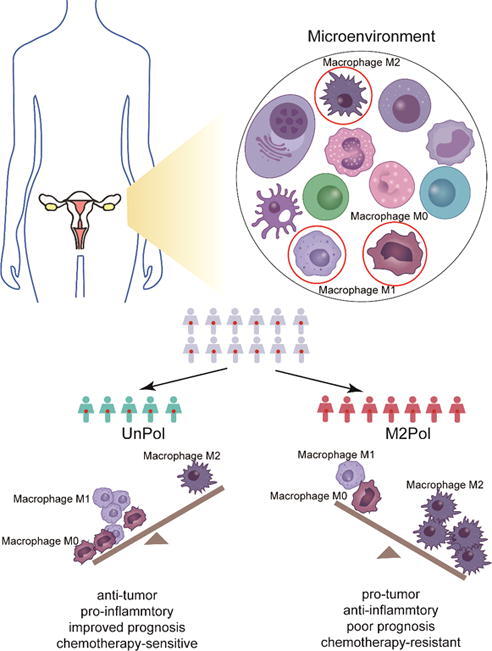

## Introduction

Ovarian cancer (OV) is the most common cause of gynecological cancer-related death, with a high incidence and mortality rate [1]. Most OV cases are at an advanced stage at the time of diagnosis. The available treatment options for patients with OV consist of surgery and carboplatin plus paclitaxel chemotherapy. However, most patients experience relapse after chemotherapy, and the median survival time for patients with recurrent OV is <2 years [1], [2], [3]. Although some progress has been made in the management of patients with OV, clinical outcomes remain unsatisfactory, due to the lack of predictive biomarkers that can accurately predict responsiveness to chemotherapy.

The tumor is a highly complex ecosystem composed of heterogeneous cancer cells, diverse infiltrating immune cells, stromal cells and vascular structures that are collectively referred to as the tumor microenvironment (TME), which significantly affects tumor onset, progression and metastasis, as well as treatment responsiveness [4], [5]. As the most abundant plastic and heterogeneous stromal cell population in the TME, tumor-associated macrophages (TAMs) have been reported to play pivotal roles in angiogenesis, tumor cell migration and anti-tumor immunity in a tissue-specific and context-dependent manner [6], [7], [8], [9]. According to the conventional binary polarization concept, tumor-infiltrating naive (M0) macrophages can either be polarized into classically or alternatively activated macrophages (referred to as M1-like or M1 and M2-like or M2, respectively) in response to different microenvironmental signals or maintained in the TME in the absence of external signals [8]. Generally, M1 macrophages have anti-tumor and pro-inflammatory functions, whereas M2 macrophages exhibit anti-inflammatory/immunosuppressive properties [10]. Macrophage differentiation is characterized by strong plasticity; indeed, the M1 and M2 macrophage phenotypes represent the extremes of a continuum, and TAMs can alternate between these different functional states in a given microenvironment [11]. Furthermore, increasing evidence also demonstrates that the activation states of macrophages display dynamic heterogeneity in the TME and play critical roles in a variety of cancer types [12], [13], [6]. However, the activation status of macrophages and its effects on biological and clinical outcomes in OV remain far from being fully characterized.

In the present study, we performed a pan-cohort characterization of the activation status of macrophages in the TME. This analysis was carried out both at the bulk-tissue and the single-cell level. The association between macrophage heterogeneity, clinical outcomes and therapeutic responsiveness was also evaluated, in the hope that these findings will gain a better understanding of the dynamic heterogeneity of macrophages in different patient groups and provide a basis for individualized management for OV.

## Methods

### Data acquisition

Bulk tumor RNA-seq data and clinical information of patients with OV were obtained from the Gene Expression Omnibus (GEO), The Cancer Genome Atlas (TCGA), ArrayExpress databases and published literature in PubMed. After excluding OV cohorts with unavailable overall survival (OS) data or chemotherapy response information for at least 40 samples, a total of 2,791 OV samples from 26 cohorts were included in the present study. The evaluation of the responsiveness to chemotherapy was determined based on the original previous studies [14], [15]. We refer to patients with complete or partial response to chemotherapy as the ‘sensitive group’. Patients with progressive or stable disease are referred to as the ‘resistant group’. Detailed information on the OV cohorts used in the present study is summarized in Supplementary Table 1.

Single-cell RNA sequencing (scRNA-seq) profiles and clinical information from five independent OV specimens were obtained from the GEO (https://www.ncbi.nlm.nih.gov/geo/query/acc.cgi?acc=GSE154600) [16], involving 167,690 single cells of two samples from chemotherapy-resistant patients, one from a chemotherapy-refractory patient and two from chemotherapy-sensitive patients.

Additionally, bulk tumor RNA-seq data and clinical information of patients treated with immune blockade therapy [anti-programmed death 1 (PD-1), anti-cytotoxic T-lymphocyte associated protein 4 (CTLA-4) and anti-programmed death ligand 1] were obtained from previous studies by Snyder et al. [17], Gide et al. [18], Hugo et al. [19] and Van Allen et al. [20].

### Data processing and quality control

Raw microarray data from Affymetrix HG-U133A and HG-U133_Plus_2 platforms were normalized using the Robust Multiarray Average algorithm [21]. Processed and normalized data from other platforms were provided by authors or other available databases. When multiple probes were mapped to the same gene, the probe with the highest signal value was used as the expression of the gene.

Quality control and downstream analysis of scRNA-seq data were conducted using the Seurat R package (version 4) [22]. Default values were used for all parameters, unless otherwise stated. The gene expression matrices of five bulk tumor samples were integrated into a Seurat object using the merge function. Quality filtering was performed to exclude low-quality cells with <200 expressed genes, unique molecular identifiers (UMIs) >20,000 and >10% mitochondrial-derived UMI counts. As a result, 43,057 cells were retained for downstream analysis.

Normalization was conducted using the SCTransform function in Seurat. The parameters “vars.to.regress” = “percent.mt”, “nCount_RNA” and “nFeature_RNA” were set in order to remove batch-associated irrelevant sources of variation, allowing the identification of 3,000 highly variable genes (HVGs). In order to identify and remove doublet events from the single-cell data, the DoubletFinder package was used. A total of 2,839 doublets (6.9% doublet rate) were removed, leaving 40,218 cells for subsequent analysis.

The RunPCA function was used to carry out principal component (PC) analysis on the HVGs. The top 30 PCs were selected for downstream analysis using the Elbowplot function. To exclude any batch effect between the samples, the RunHarmony function was used. Subsequently, cells were divided into multiple clusters using the FindNeighbors function (reduction = “harmony”) and the FindClusters function (resolution = 1), then visualized with UMAP plots [23]. The cell types were identified using markers from a previous study [24] and from the CellMarker database [25].

### Inference of tumor-infiltrating immune cell abundance

In order to evaluate the distribution of specific cellular components of the immune microenvironment, CIBERSORT was used to determine the relative proportion of 22 immune cell types [26]. To avoid systematic bias between multiple cohorts, the relative proportion of the 22 immune cell types was determined separately in each cohort, and subsequently pooled for further analysis.

### Consensus clustering

Consensus clustering was carried out using K-means clustering based on Pearson’s correlation coefficient. The clustering method based on the Euclidean distance metric was used in this analysis, which was executed with the “ConsensusClusterPlus” R package by selecting 80% patient and 100% feature [27]. Cluster numbers, K were assumed to range from 2 to 8, and the cumulative distribution function (CDF) and silhouette width were used to evaluate the relative change by increasing the number of clusters. The flatter the middle segment of the CDF curve, the less ambiguous the sample is (expressed using the proportion of ambiguous clustering value). The larger the silhouette width, the better the clustering effect [28].

### Single-sample gene set enrichment analysis (ssGSEA)

Immunoregulatory genes were obtained from a previous study [29], and hallmark gene sets were retrieved from the Molecular Signatures Database [30]. The enrichment scores of the immunoregulatory gene sets or hallmark gene sets for each sample were calculated using ssGSEA with the GSVA R package [31].

### Development of the macrophage polarization-derived artificial neural network model (MacroANN)

Neural networks are machine-learning models based on input layer, hidden and output layers. Moreover, it is a widely used method for regression analysis and classification of high-dimensional data. In order to capture and quantify the infiltration pattern of macrophage subtypes, the infiltration abundance of each macrophage subtype was converted into feed-forward neural networks using the nnet package. We then developed a machine-learning model, MacroANN, using a neural network-based classifier that distinguishes the UnPol from the M2Pol functional state. Samples were randomly divided into a training cohort (60%) and a testing cohort (40%). We predicted labels of the testing cohort and recorded the difference in prognosis/chemotherapy response. This classifier was also applied to the immunotherapy cohorts to distinguish the patients likely to benefit from treatment from those who may not benefit from treatment.

### Immunohistochemistry

Ovarian cancer tissues of 17 patients who underwent surgical resection were obtained from Harbin Medical University Cancer Hospital (Harbin, China). All patients intravenously received 6 to 8 cycles of platinum-based combination chemotherapy (either cisplatin/carboplatin/nedaplatin plus paclitaxel or carboplatin/nedaplatin plus docetaxel or nedaplatin plus paclitaxel liposome) with an interval of 21 days after the primary surgery. This study was approved by the Clinical Research Ethics Committee of Harbin Medical University Cancer Hospital (KY2021-45) and all patients provided written informed consent prior to the study. The response to chemotherapy was evaluated based on Gynecologic Oncology Group criteria [32]. Platinum-resistant disease was defined as a progression or recurrence after a platinum-free interval of <6 months.

Immunohistochemical (IHC) staining was performed in paraffin-embedded tissue. Serial sections (4 mm thick) were placed on slides coated with 3-aminopropyltriethoxysilane. IHC was performed using a two-step protocol. Paraffifin sections were first deparaffifinized and hydrated. After microwave antigen retrieval and neutralization of endogenous peroxidase, slides were preincubated with blocking serum and then incubated overnight with primary antibodies (anti-CD86 antibody, Abcam; anti-CD206 antibody, Abcam). Subsequently, the sections were serially rinsed, incubated with second antibodies. Positive staining was visualized with DAB (3, 3-diaminobenzidine), then counterstained with hematoxylin. IHC staining of the TMA sections was evaluated manually in randomly five high power fields (HPFs) under the microscope (Leica DM4 P; Leica, Wetzlar, Germany). The staining intensity was evaluated as follows: 0 (negative), 1 (weak), 2 (moderate) and 3 (strong). The percentage of staining was scored as follows: 0 (0%), 1 (0–25%), 2 (25–50%), 3 (50–75%) and 4 (75–100%). Finally, the expression levels of CD86 and CD206 were obtained using the immunoreactive score (the staining intensity × the staining percentage) as previously described [33].

### Statistical analysis

All analyses were performed using R version 3.6. Statistical differences between the two groups of continuous variables were determined using two-tailed Wilcoxon rank-sum test. The χ^2^ test was used for two groups of categorical variables. P < 0.05 was considered statistically significant. Meta-analysis was performed using the R package “meta.” A random-effect model was employed to the *meta*-analysis, if there was a high degree of heterogeneity (I^2^ > 50%), otherwise, a fixed-effect model was employed. The optimal cut-off value for the immunotherapy cohort was selected using the survminer package. For other undeclared states, the median was used as the cut-off value. Univariate and multivariate Cox proportional hazards regression analyses were used to evaluate the association between overall survival and clinical variables using the coxph function of the R survival package. The hazard ratios (HR) and 95% confidence intervals (CI) were calculated.

## Results

### Immunogenomic characterization of public OV datasets reveals critical roles of macrophages in OV prognosis and chemotherapy

We first quantitatively estimated the cellular composition of immune infiltrates in the TME and evaluated the prognostic impact of each immune cell type in 18 publicly available OV cohorts with overall survival information using univariate Cox regression analysis. A *meta*-analysis was then used to leverage the 18 OV cohorts for an overall prognostic evaluation for each immune cell type.

**Fig. 1:**
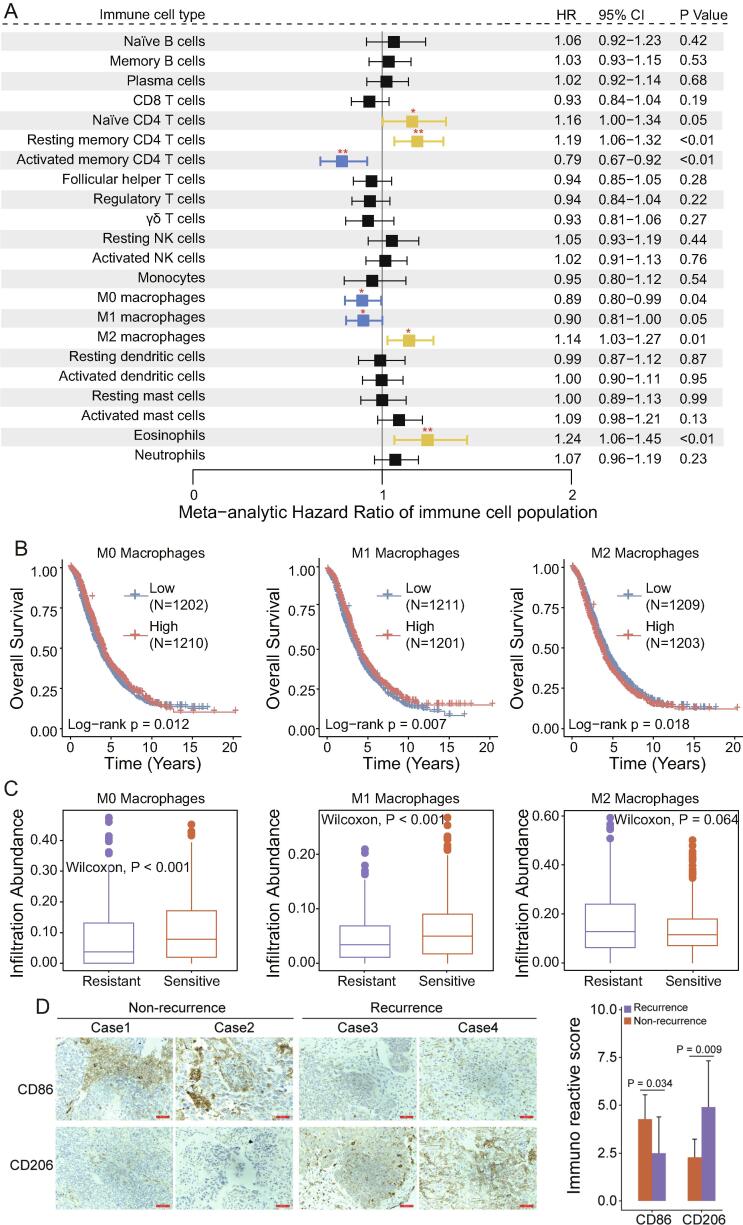
**Association of the cellular composition of the immune infiltrates with prognosis and responsiveness to chemotherapy.** (A) Meta-analysis of infiltration abundance data of each immune cell type using univariate Cox regression in 18 ovarian cancer cohorts. The data are presented as forest plots showing the HR and 95% CI. The high vs. low groups were obtained according to the median. (B) Kaplan-Meier survival curves of patients with ovarian cancer and different immune infiltrates. (C) Infiltration abundance of each immune cell type in chemotherapy-sensitive patients and chemotherapy-resistant patients. (D) Representative IHC staining of CD86, CD206 in recurrence and non-recurrence patients. Expression of CD86 and CD206 in human recurrence and non-recurrence patients, respectively. The data were analyzed using Student's *t* test. HR, hazard ratio; CI, confidence interval; IHC, immunohistochemistry.

The pooled results indicated that the infiltration of naïve CD4 T cells (HR = 1.16; 95% CI, 1.00–1.34; P = 0.045), resting CD4 memory T cells (HR = 1.19; 95% CI, 1.06–1.32; P = 0.002), M2 macrophages (HR = 1.14; 95% CI, 1.03–1.27; P = 0.014) and eosinophils (HR = 1.24; 95% CI, 1.06–1.45; P = 0.006) was associated with poor prognosis. By contrast the infiltration of activated CD4 memory T cells (HR = 0.79; 95% CI, 0.67–0.92; P = 0.003), M0 macrophages (HR = 0.89; 95% CI, 0.80–0.99; P = 0.04) and M1 macrophages (HR = 0.90; 95% CI, 0.81–1.00; P = 0.05) was associated with improved prognosis (Fig. 1A and Supplementary Fig. 1). Survival analysis showed that patients with low infiltration of naïve CD4 T cells, resting memory CD4 T cells, M2 macrophages or eosinophils had a significantly longer OS than those with high infiltration, whereas patients with low infiltration of activated CD4 memory T cells, M0 macrophages or M1 macrophages had a significantly shorter OS than those with high infiltration (Fig. 1B and Supplementary Fig. 2).

Next, we further examined the association between each immune cell type with responsiveness to chemotherapy in 11 public OV cohorts with available chemotherapy information. The infiltration abundance of nine immune cell types was significantly different between the chemotherapy-sensitive and chemotherapy-resistant patients (Fig. 1C and Supplementary Fig. 3). Memory B cells, follicular helper T cells, M0 macrophages, M1 macrophages, activated dendritic cells and γδ T cells were significantly more abundant in the chemotherapy-sensitive patients. In contrast, infiltration abundance of naïve B cells, resting NK cells, and resting CD4 memory T cells were increased in the chemotherapy-resistant patients. To further validate the clinical significance of macrophages in OV, we conducted an immunohistochemistry assay to investigate the expression of M1 macrophage marker (CD86) and M2 macrophage marker (CD206) of 17 OV patients (including 10 patients with recurrence and 7 patients without recurrence). Representative images were shown in Fig. 1D. As shown in Fig. 1D, the expression level of CD86 in non-recurrent patient tissues is significantly higher than recurrent patient tissues (P = 0.034), whereas the expression level of CD206 in non-recurrent patient tissues is significantly lower than recurrent patient tissues (P = 0.009) (Fig. 1D). These results indicated that the functional complexity of macrophages is associated with prognosis and therapeutic efficacy in OV.

### Single-cell transcriptome analysis confirms the association between the functional state of macrophages and responsiveness to chemotherapy

**Fig. 2:**
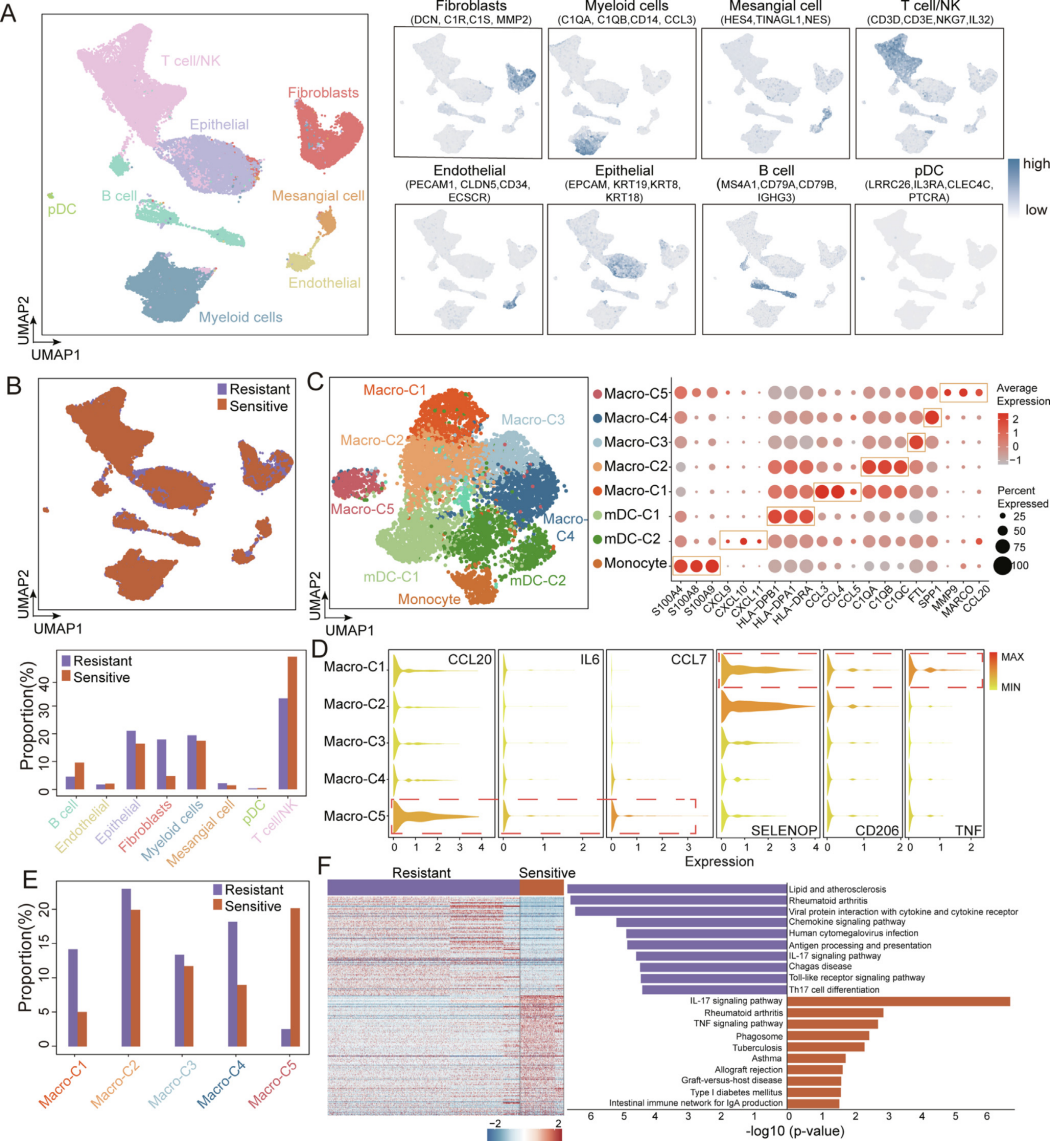
**Single-cell RNA sequencing analysis of intra-tumoral cell type heterogeneity in ovarian cancer.** (A) UMAP visualization of 40,218 single cells, color-coded by cell type (left) or according to the expression of cell-type specific gene markers (right). (B) UMAP visualization and frequency of each cell type in samples from the resistant (purple) and sensitive (orange) groups. (C) UMAP plot of myeloid cells, color-coded by cluster (left). Dotplot of markers of each cell clusters (right). The size of the dots is proportional to the abundance, the color represents the expression levels. (D) Violin plots of the gene markers in five macrophage clusters. (E) Frequency of macrophage cell types in samples from the resistant (purple) and the sensitive (orange) groups. (F) Heatmap and Kyoto Encyclopedia of Genes and Genomes pathway analysis of differentially expressed genes in macrophages from resistant vs. sensitive samples. The top 10 upregulated pathways are shown for the resistant (purple) and the sensitive (orange) groups. pDC, plasmacytoid dendritic cell; mDC; myeloid dendritic cell; NK, natural killer.

We then examined scRNA-seq data from 5 patients with OV. After quality control and exclusion of any batch effect, 40,218 single cells were analyzed, including 31,358 single cells originating from chemotherapy-resistant patients and 8,860 from sensitive patients. The 40,218 single cells were clustered into eight populations. Using cell-specific markers, we identified four immune cell types, namely T/natural killer (NK) cells, B cells, myeloid cells and plasmacytoid dendritic cells, along with four non-immune cell types (fibroblasts, as well as mesangial, endothelial and epithelial cells) (Fig. 2A). Chemotherapy-resistant samples were highly enriched in fibroblasts, myeloid cells and epithelial cells, whereas B and T/NK cells were overrepresented in the chemotherapy-sensitive samples (Fig. 2B).

Subsequently, myeloid cells were analyzed separately. Myeloid cells were further grouped into five macrophage subclusters (macro-C1, -C2, -C3, -C4 and -C5), two myeloid dendritic cell subclusters and one monocyte subcluster (Fig. 2C). Macro-C1 specifically expressed chemokine genes [C-C chemokine ligand 3 (CCL3), CCL4 and CCL5] and displayed co-expression of both M1 (TNF) and M2 (CD206 and selenoprotein P) macrophage markers, suggesting a complex polarization phenotype (Fig. 2C and D). Macro-C2 demonstrated increased expression levels of complement C1q, which may drive TAMs to M2 polarization. Macro-C3 demonstrated increased expression levels of FTL, which has been shown to exert both pro-proliferative and pro-angiogenic effects in the results of previous studies [34]. Moreover, macro-C4 expressed high levels of secreted phosphoprotein 1 (SPP1), a marker of M2 macrophages associated with tumor angiogenesis. Macro-C5 exhibited high expression levels of M0 macrophage markers, corresponding to a resting macrophage phenotype. In addition, the macro-C5 subcluster expressed high levels of pro-inflammatory cytokines and chemokines (CCL7, CCL20 and IL6; Fig. 2D), suggesting polarization towards the M1 phenotype. These results indicated that macro-C2, -C3 and -C4 clusters correspond to M2-like macrophage, and macro-C5 cluster corresponds to M1-like macrophages.

Furthermore, we also observed differences in the distribution of macrophage subclusters. The M2-like macrophages (macro-C2, -C3 and -C4 clusters) were predominant in the chemotherapy-resistant patients, whereas samples from the chemotherapy-sensitive patients were enriched in the macro-C5 cluster (Fig. 2E). Subsequently, hierarchical clustering and KEGG pathway enrichment analysis were carried out on the macrophage subclusters of the chemotherapy-resistant and chemotherapy-sensitive samples, and distinct expression patterns were observed (Fig. 2F). Macrophage subclusters from the chemotherapy-resistant group expressed genes involved in multiple disease-associated pathways (such as ‘lipid and atherosclerosis’ and ‘rheumatoid arthritis’). By contrast, macrophage subclusters derived from the chemotherapy-sensitive group were enriched in immune and anti-tumor-related pathways (‘IL-17 signaling pathway’ and ‘TNF signaling pathway’). Thus, the scRNA-seq analysis supported the findings of the bulk tissue transcriptomics and suggested that the activation status of macrophages is associated with responsiveness to chemotherapy.

### Functional states of macrophages identify clinically distinct patient subgroups

**Fig. 3:**
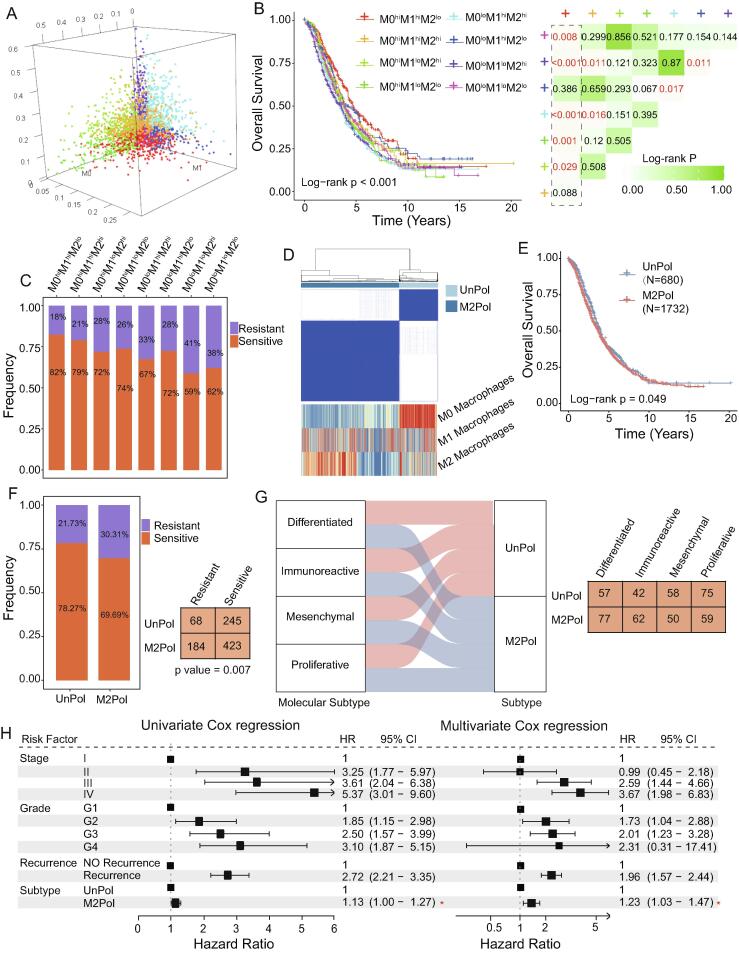
**Identification of potential macrophage subtypes of ovarian cancer.** (A) 3D scatter plot showing the frequency of tumor-infiltrating M0 (x-axis), M1 (y-axis) and M2 macrophages (z-axis) in each ovarian cancer sample. (B) Kaplan-Meier curves of the overall survival of patients stratified according to the relative abundance of the tumor-infiltrating macrophage subtypes. (C) The proportion of chemotherapy-resistant and -sensitive patients among eight patient groups stratified according to their distribution of M0, M1 and M2 macrophages. (D) Consensus clustering matrix of ovarian cancer samples. (E) Kaplan-Meier curves of the overall survival of patients in the UnPol and M2Pol subtypes. (F) The proportion of chemotherapy-resistant and -sensitive patients in the UnPol and M2Pol groups. The data were analyzed using a χ^2^ test. (G) Alluvial diagram showing the association between macrophage subtypes and TCGA molecular subtypes. (H) Univariate and multivariate analyses of the association between macrophage subtypes and other clinical characteristics. HR, hazard ratio; CI, confidence interval.

We then explored the relationship between the relative infiltration abundance of different macrophage subtypes and patient prognosis. A total of 2,791 OV samples were divided into eight categories according to the median infiltration abundance of M0, M1 and M2 macrophages (Fig. 3A). We observed significant differences in OS among the eight groups (P = 0.0005; log-rank test). Patients in the M0^hi^M1^hi^M2^lo^ group demonstrated superior OS compared with the other groups (Fig. 3B).

Furthermore, the effect of macrophage infiltration on the overall response rate was analyzed. The results demonstrated that patients in the M0^hi^M1^hi^M2^lo^ group had the highest response rate to chemotherapy (82%), whereas patient in the M0^lo^M1^lo^M2^hi^ group had the lowest (59%) (Fig. 3C). An unsupervised consensus clustering analysis enabled the identification of two distinct patient subgroups with different functional states of macrophages (Fig. 3D). As shown in Fig. 3D, cluster 1 was characterized by high M0 and M1 macrophage abundance (referred to as the UnPol subtype thereafter), whereas cluster 2 displayed high abundance of M2 macrophages and relatively low levels of M0 and M1 macrophages (referred to as M2Pol).

Survival analysis indicated that the UnPol subtype was associated with improved OS compared with the M2Pol subtype (P = 0.049; log-rank test; Fig. 3E). It was also noted that the UnPol subtype was distinctly different from the M2Pol subtype in terms of chemotherapy response rate (P = 0.007; χ^2^ test; Fig. 3F). In addition, the TCGA-defined molecular subtypes (differentiated, immunoreactive, mesenchymal and proliferative) could be almost equally divided into the UnPol and M2Pol subtypes identified using our approach, which demonstrates the practicality of these two subtypes (Fig. 3G). Furthermore, this macrophage-based subtype classification maintained a significant association with OS (HR = 1.23; 95% CI, 1.03–1.47; P = 0.024; multivariate Cox regression) following adjustment for other clinical features through multivariate Cox regression analysis (Fig. 3H). Altogether, these results suggested that macrophage polarization in the TME significantly distinguishes the clinical outcomes of patients with OV.

### Functional states of macrophages distinguish immunologically distinct subgroups

**Fig. 4:**
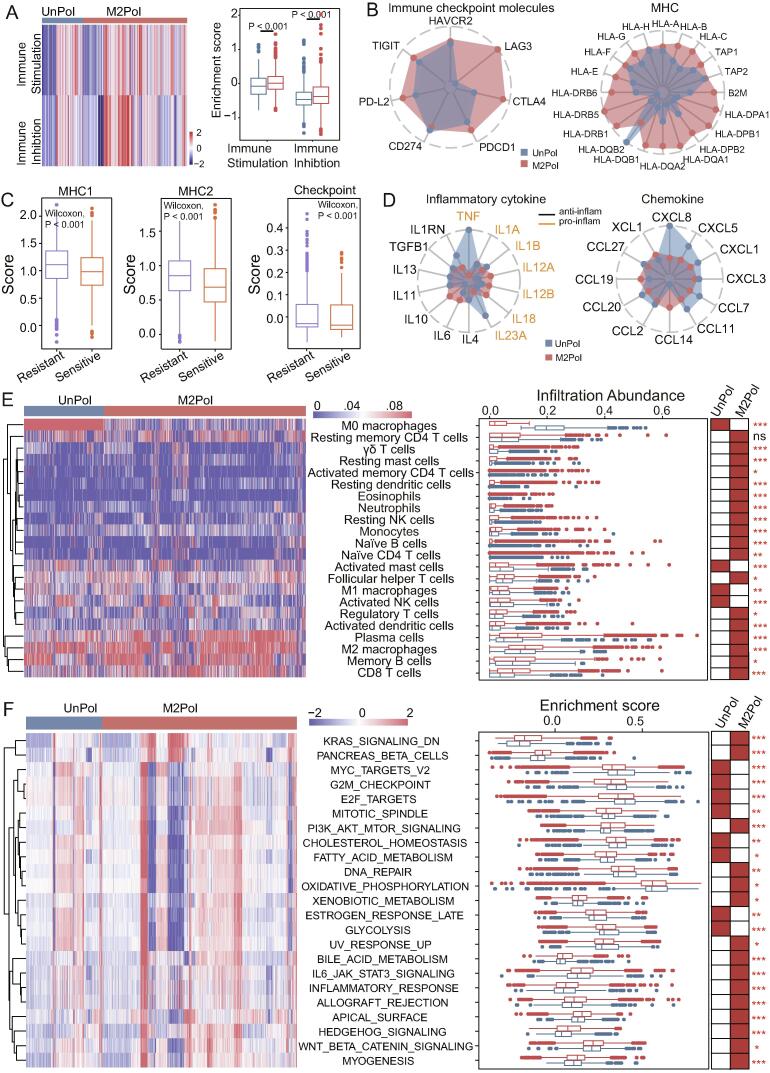
**Biological and clinical characteristics of UnPol and M2Pol subtypes.** (A) Heatmap and boxplot of the immune stimulation and immune inhibition enrichment score in the UnPol and M2Pol subtypes. The data were analyzed using two-sided Wilcoxon rank-sum tests. (B) Radar chart depicting the expression of immune-checkpoint and MHC molecules in the UnPol and M2Pol subtypes. (C) Boxplot showing the expression levels of MHC and immune-checkpoint molecules in samples from the chemotherapy-sensitive and chemotherapy-resistant groups. (D) Radar chart depicting the expression of pro-inflammatory cytokines and chemokines in the UnPol and M2Pol subtypes. Pro-inflammatory cytokines are represented in yellow font. Black font denotes the anti-inflammatory cytokines. (E) Heatmap of the infiltration abundance of different immune subpopulations. (F) Heatmap of the enrichment scores of cancer hallmark pathways for each sample in two subtypes.

We further explored the functional states of macrophages that may lead to differences in the immunological behavior of the subgroups. First, we obtained a set of 63 immunoregulatory genes from the study by Vidotto et al. [29], including both immune activators and inhibitors, then calculated ssGSEA scores to evaluate the degree of immunological activation or inhibition. As shown in Fig. 4A, the M2Pol subtype displayed higher immune stimulation (P = 1.6 × 10^−13^; Wilcoxon rank-sum test) and immune inhibition (P = 6.8 × 10^−9^; Wilcoxon rank-sum test) compared to UnPol subtype, suggesting that different mechanisms may contribute to tumor-immune escape between two subtypes.

Mechanisms of tumor escape from the immune system can involve tumor-intrinsic and -extrinsic factors. Tumor-intrinsic factors of immune escape include tumor immunogenicity, as well as the expression of immune checkpoint molecules. However, there was no significant difference in tumor immunogenicity between the UnPol and the M2Pol subtypes, as evidenced by the similar tumor mutation burden, homologous recombination deficiency and neoantigen loads in the TCGA data (Supplementary Fig. 4). Moreover, we analyzed the average expression levels of immune checkpoint molecules and found higher expression in the M2Pol compared with the UnPol subtype (Fig. 4B). Major histocompatibility complex (MHC) class I and II, as well as non-classical MHC molecules, which are antigen-presenting molecules, were also upregulated in the M2Pol subtype (Fig. 4B). The expression levels of MHC and immune checkpoint molecules were also analyzed in the single-cell transcriptome data from the chemotherapy-resistant and -sensitive patients. It was observed that macrophages expressed significantly higher levels of MHC and immune checkpoint molecules in the resistant compared with the sensitive patients (Fig. 4C). The difference in the expression of antigen-presenting molecules was related to T cells and macrophages. These results demonstrated that the M2Pol subtype tumors expressed immune checkpoint molecules to escape from immune surveillance.

Previous studies have identified the absence of immune cells, the presence of immunosuppressive cells, fibrosis and the release of immunosuppressive cytokines in the TME as tumor-extrinsic factors that promote escape from the immune system [35], [36], [37]. The expression levels of pro-inflammatory cytokines and chemokines were examined in the UnPol and M2Pol subgroups. Pro-inflammatory cytokines were preferentially expressed in the UnPol subtype, whereas the expression of anti-inflammatory cytokines was predominant in the M2Pol subtype (Fig. 4D). Chemokine expression is variable in different states, using chemotactic factors for macrophage populations in the tumor (Fig. 4D). The tumor suppressor CCL14 was upregulated in the M2Pol subtype. By contrast, the pro-inflammatory chemokines CXCL5 and CXCL1 were upregulated in the UnPol subtype.

To evaluate the extrinsic immune evasion factors specifically, we compared the distribution and relative abundance of different immune cell subpopulations in the TME between the UnPol subtype and the M2Pol subtype. As shown in Fig. 4E, immunosuppressive cell subpopulations were enriched in the M2Pol subtype, suggesting that an increase in immunosuppressive cells might contribute to the immune escape of the M2Pol subtype.

To further examine whether tumors in the UnPol and M2Pol subtypes presented different biological features, we calculated the ssGSEA scores of hallmark gene sets for each sample and identified differentially expressed gene modules involved in the cell cycle, inflammation, immune responses and cellular metabolism (Fig. 4F). The UnPol subtype was characterized by high activation of cell cycle pathway, while the M2Pol subtype had a higher inflammation/immune response. Increasing studies pointed that cellular metabolism play critical role in the function of cancer cells and immune cells, which also manifest differently across different phenotypes [38], [39], [40]. ‘Oxidative phosphorylation’ and ‘glycolysis’ hallmark gene set, which were the main ways of producing energy in the form of adenosine triphosphate (ATP), were enriched in the M2Pol subtype and UnPol subtype, respectively. Acetyl-CoA from glycolysis promotes fatty acid synthesis, which is required for membrane remodeling and the production of inflammatory mediators and tend to be predominant in the UnPol subtype. Previous studies have suggested that the ‘fatty acid metabolism’ and ‘cholesterol homeostasis’ are essential for energy homeostasis and metabolic health [41], [42]. However, bile acid synthesis, an important pathway for cholesterol, may inhibit the production of pro-inflammatory cytokines by macrophages [43].

### MacroANN predicts patient outcomes and therapeutic efficacy

**Fig. 5:**
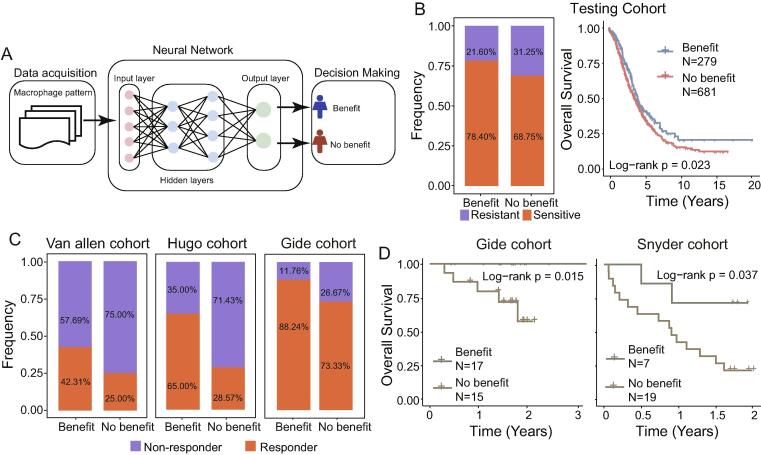
**MacroANN is a prognostic factor and predictive biomarker for therapeutic efficacy.** (A) Schematic overview of MacroANN. (B) Rate of clinical response to chemotherapy and Kaplan- Meier curves for patients in the benefit and no-benefit groups in the testing cohort. (C) Rate of clinical response to immune blockade therapy in the benefit and non-benefit groups in the different cohorts. (D) Kaplan-Meier survival curves showing overall survival of patients receiving immune blockade therapy in the benefit and non-benefit groups in the different cohorts.

Considering the association between the activation of macrophages and tumor biological and clinical behavior, a macrophage polarization-derived artificial neural network model (referred to as MacroANN) was developed in the training cohort to predict clinical outcome and therapeutic efficacy (Fig. 5A). When applied to the testing cohort, MacroANN distinguished between patients who did or not benefit from chemotherapy, with response rates of 78.40 and 68.75% in the benefit and the no-benefit groups (Fig. 5B). Patients in the benefit group experienced significantly improved survival time than those in the no-benefit group (P = 0.023; log-rank test; Fig. 5B). The 5-year OS of the patients in the benefit group was 38.13%, whereas that of the no-benefit group was 29.20%.

To further test whether MacroANN provided additional predictive value for responsiveness to immunotherapy, MacroANN was applied to three immunotherapy cohorts. The model yielded accurate predictions regarding clinical benefit and divided the patients into a benefit and a no-benefit group. A higher response rate was observed in patients in the predicted benefit group compared with those in the no-benefit group. The response rates of the patients in the benefit group were 42.31, 65 and 88.24%, in the Van Allen, Hugo and Gide cohorts, respectively. These were higher than the corresponding response rates (25, 28.57 and 73.33%, respectively) for the patients in the no-benefit group (Fig. 5C). Moreover, the results from a survival analysis suggested that the predicted benefit group was significantly associated with improved OS (P = 0.015 for the Gide cohort and P = 0.037 for the Snyder cohort; log-rank test; Fig. 5D). These results suggest that MacroANN can predict clinical outcomes, as well as responsiveness to chemotherapy and immunotherapy.

## Discussion

OV is a heterogeneous disease characterized by biological, cellular and molecular features, posing critical clinical challenges for management and treatment [14]. The accumulated efforts to characterize the TME have uncovered the complexity and diversity of its immunological components, which have been proposed as potential prognostic and therapeutic markers for patients with OV [44], [45], [46]. TAMs are a predominant population and critical mediators in TME, and the prognostic value of their phenotypes has already been demonstrated in several previous studies, revealing inconsistent and controversial results. Increasing evidence has also demonstrated that macrophage phenotypes represent a continuum of phenotypic and functional states that continuously evolve during cancer progression and metastasis [47]. However, how the diversity of macrophage subtypes and their homeostasis may affect the biological and clinical behavior of OV remains unclear.

In the present study, using large-scale, bulk RNA-seq and single-cell RNA-seq data, we systematically investigated the association between macrophage activation states and prognosis, as well as responsiveness to chemo- and immunotherapy. The pan-cohort results suggested that high infiltration of M0 and M1 macrophages was associated with improved outcome and therapeutic efficacy, whereas the opposite was true for M2 macrophages. These findings are in line with previous studies suggesting that M2 macrophages accelerated the progression and metastasis of OV and that patients with OV and a prevalence of M1 macrophages experienced prolonged OS [48], [49], [50]. However, most of these studies have solely focused on the infiltration abundance of M1 or M2 TAMs, and only a few studies have evaluated the role of M0 macrophages. Furthermore, recent studies have also indicated that the subtypes and prognosis of OV are strongly associated with the relative immune infiltration rather than the absolute infiltration of immune cells. For example, a study involving 112 patients with OV found that higher M1/M2 TAM ratios were associated with improved 5-year survival using immunohistochemistry and immunofluorescence methods [51]. Using unsupervised consensus clustering analysis, our study identified two novel subtypes (UnPol and M2Pol) related to heterogeneous TAM activation. These two subtypes extended beyond the classic molecular subtype based on gene expression and were associated with the clinical outcome and responsiveness to chemotherapy, independently of clinicopathological variables.

High expression of pro-inflammatory cytokines and lack of immunosuppressive cells were two of the most notable biological characteristics of the UnPol subtypes. The distinct biological characteristics of the M2Pol subtype included increase in immunosuppressive cells and anti-inflammatory cytokines. Furthermore, the M2Pol subtype also exhibited high expression of immune checkpoint molecules, which may potentially be the underlying cause of the poor prognosis. However, MHC molecules were upregulated in the M2Pol subtype. This finding suggested that MHC molecules perform different functions in different immune cells. The T cell receptor of a CD4 or CD8 T cell recognizes antigen on MHC class II or MHC class I, respectively [52], [53]. Cancer cells can escape from immune recognition by downregulating the expression of MHC class I. However, Weissman et al. demonstrated that the leukocyte immunoglobulin like receptor B1 protein on the surface of macrophages could partially bind to MHC-class I [54]. This inhibited the phagocytosis of cancer cells by macrophages and promoted tumor escape from the immune system [54]. Loss of MHC class I expression has been identified as a mechanism underlying resistance to anti-PD-1 treatment [55]. This could explain the association of the M2Pol subtype with clinical benefits from immunotherapy. In addition, several studies have revealed that CD4 T cell activation through MHC class II antigens can overcome the resistance of anti-PD-1+ anti-CTLA-4 immune checkpoint therapy [56].

KEGG enrichment pathway analysis suggested that the M2Pol subtype may be associated with KEGG terms relating to inflammation and immune responses. Numerous studies have shown that inflammation and inflammatory mediators promote the occurrence of ovarian tumors [57]. Previous studies have highlighted the importance of metabolism in defining macrophage phenotype and function [58], [40]. For example, glycolysis can be induced by different factors to exert anti-tumor or tumor-promoting effects. Glycolysis can produce reactive oxygen species, which are essential for the phagocytic activity of M1 macrophages. M2 macrophages rely on oxidative phosphorylation for metabolism.

Considering the association of macrophage activation with tumor biological and clinical behaviors, we developed an artificial neural network model to simulate the dynamic activation of macrophages. The prognostic and predictive value of this model was evaluated in different cohorts. Using this model, it was possible to stratify patients into two risk groups with different OS. Furthermore, this model may be applied in order to predict responsiveness to chemotherapy or immunotherapy.

Some limitations in our study should be acknowledged. First, only three major macrophage subtypes (M0, M1 and M2) were studied, and other macrophage subsets should be further investigated at the single-cell level. Second, there was no available OV dataset treated with immune blockade therapy. Therefore, the predictive value of this model for the response to immune checkpoint inhibitor treatment requires further examination. In addition, spatial and temporal differences among source samples will affect the analysis and should be considered in the further study.

In summary, we performed an integrated immunogenomic analysis of single-cell and bulk tissue RNA-seq data from patients with OV and defined two novel subtypes characterized by differences in macrophage phenotypes. These two macrophage-driven subtypes were associated with prognosis and therapeutic efficacy, as well as biological and immunological features that may shape the TME. We also developed an artificial neural network model to simulate the dynamic activation of macrophages, which could serve as a robust prediction tool for the responsiveness of patients with OV to chemotherapy or immunotherapy. These findings provide novel insight into macrophage heterogeneity and its association with OV prognosis. This study also highlighted the future clinical potential for tumor prevention and treatment based on macrophage activation.

## Consent for publication

All authors agree with the content of the manuscript.

## Availability of supporting data

The datasets used and/or analyzed during the present study are available from the corresponding author on reasonable request.

## Funding

This study was supported by the 10.13039/501100004731Zhejiang Provincial Natural Science Foundation of China (Grant No. LY22C060001). The funders had no roles in study design, data collection and analysis, decision to publish, or preparation of the manuscript.

## Compliance with Ethics Requirements

This study was approved by the Clinical Research Ethics Committee of Harbin Medical University Cancer Hospital (KY2021-45) and all patients provided written informed consent prior to the study.

## Declaration of Competing Interest

The authors declare that they have no known competing financial interests or personal relationships that could have appeared to influence the work reported in this paper.
