# Supplementary material for: Integrated immunogenomic analysis of single-cell and bulk tissue transcriptome profiling unravels a macrophage activation paradigm associated with immunologically and clinically distinct behaviors in ovarian cancer
Source: J Adv Res. 2022 Apr 15;44:149–60. doi: 10.1016/j.jare.2022.04.006 (PMC9936412; doi:10.1016/j.jare.2022.04.006)
Supplement: Supplementary data 1 [file mmc1.docx]

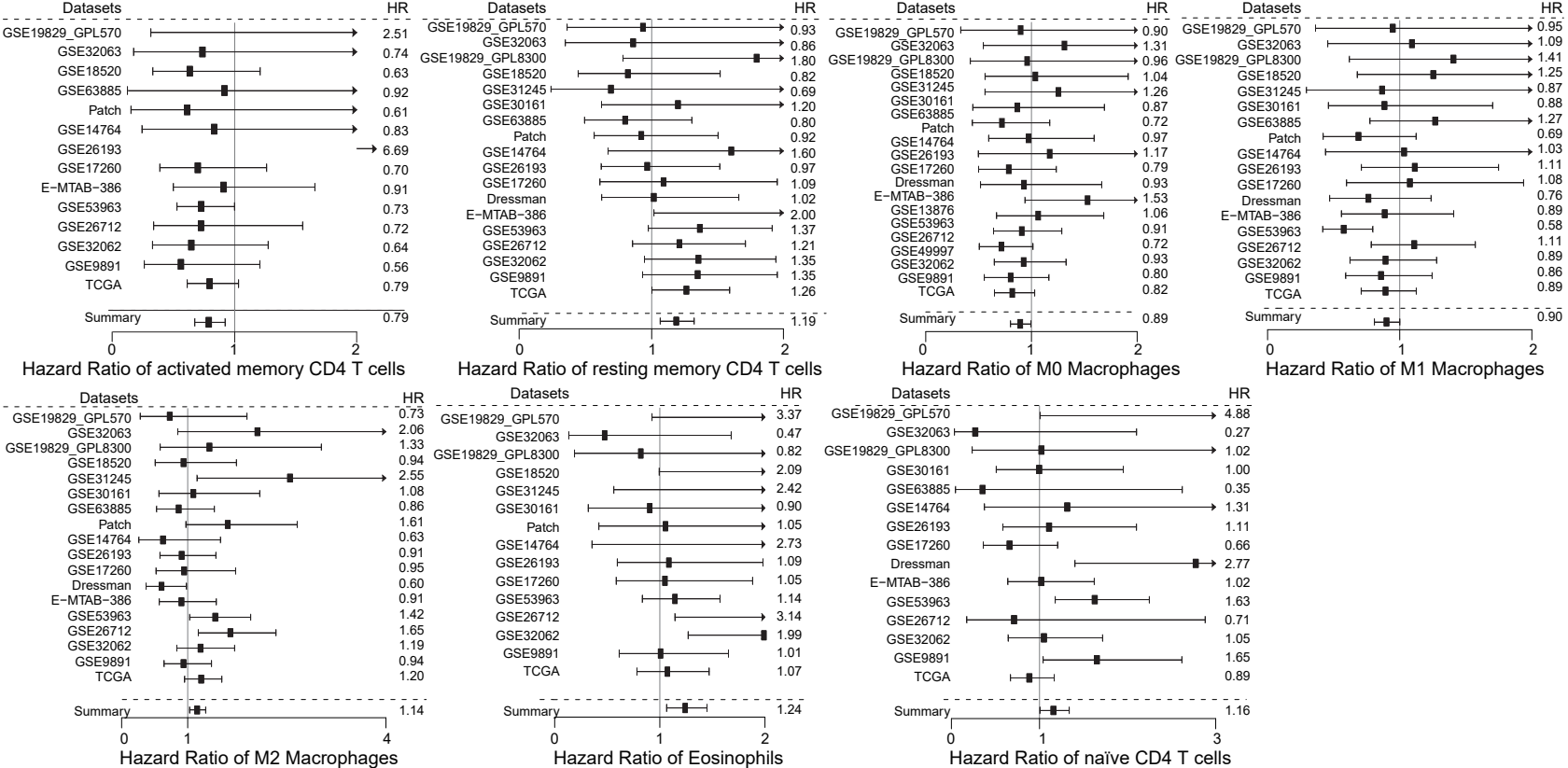


**Supplementary figure 1. Analysis of infiltration abundance data of seven immune cell type using univariate Cox regression in 18 ovarian cancer cohorts.** The data are presented as forest plots showing the HR and 95% confidence interval. The high vs. low groups were obtained according to the median. HR, hazard ratio.


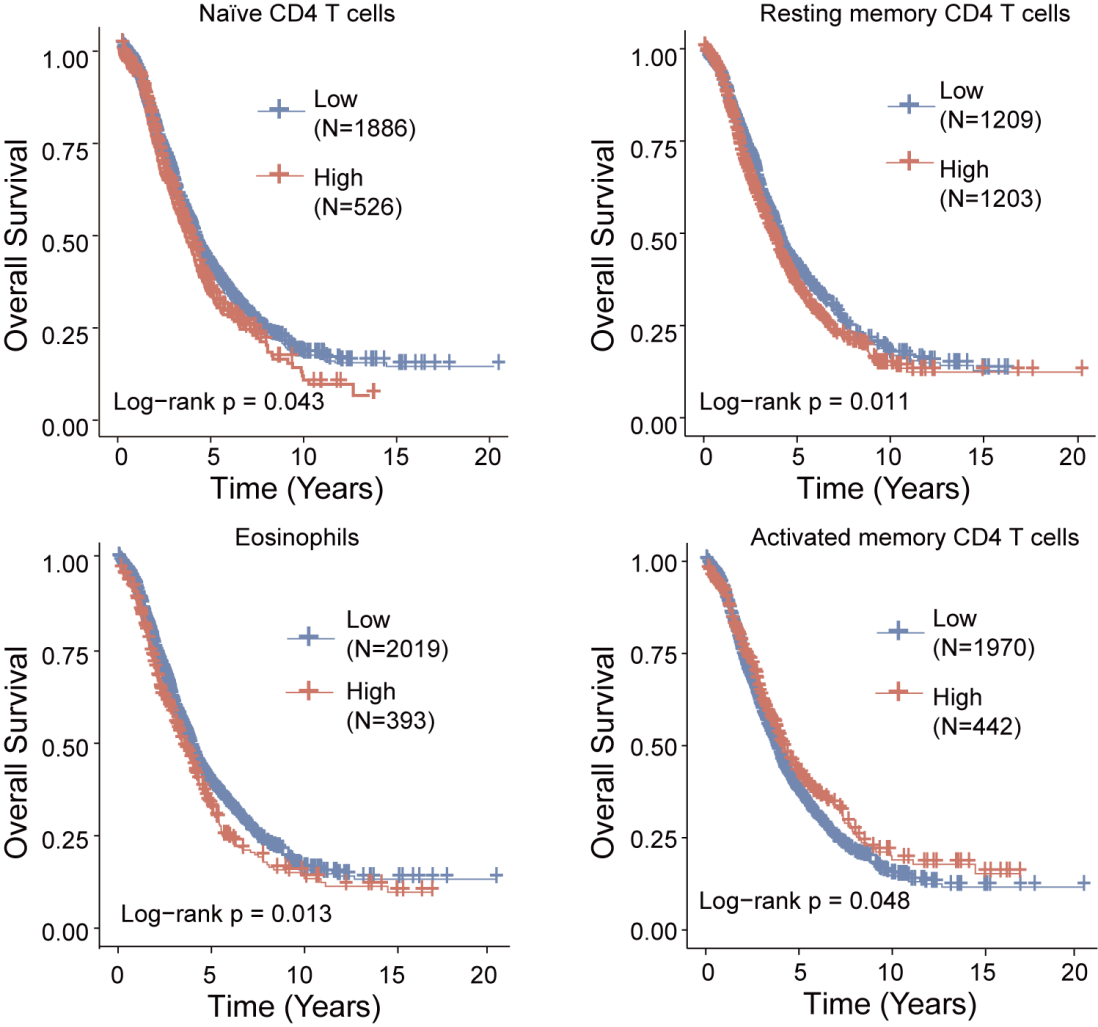


**Supplementary figure 2. Kaplan-Meier survival curves of patients with ovarian cancer stratified according to low or high infiltration of different CD4 T cell subpopulations and eosinophils.**


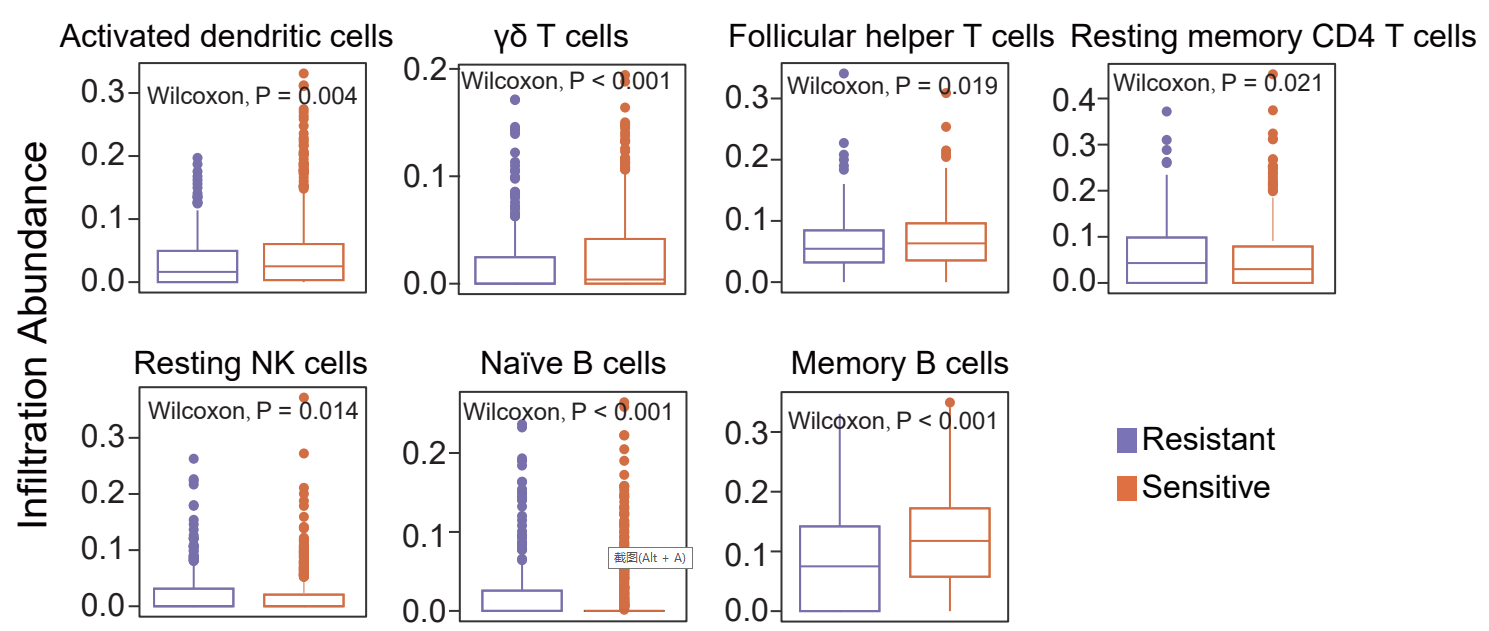


**Supplementary figure 3. Boxplot showing infiltration abundance in the chemotherapy-sensitive patients and -resistant patients.**


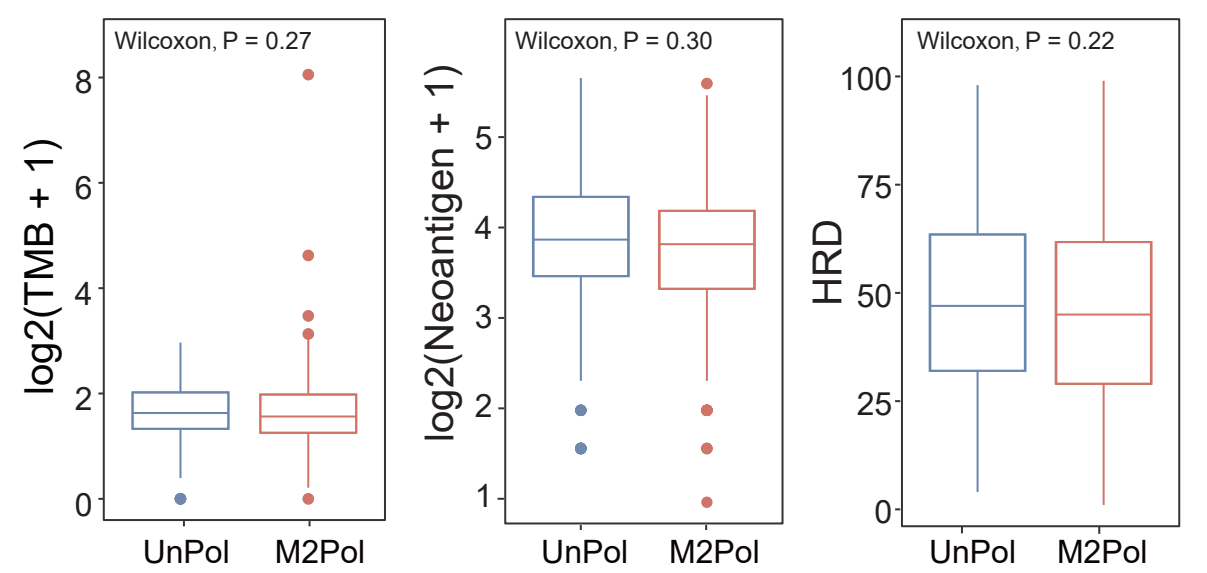


**Supplementary figure 4. Boxplots showing the tumor mutation burden distribution, homologous recombination deficiency and neoantigen load in the M2Pol and UnPol subtypes in The Cancer Genome Atlas cohort.**

**Table S1 Detailed information of ovarian cancer cohorts used in this study**

| **Datasets** | **Platform** | **Number of samples** | **Information** | **Ref** |
| --- | --- | --- | --- | --- |
| **TCGA** | **HGU133A** | **557** | **OS, chemo-response** | **21720365** |
| **GSE3149** | **HGU133A** | **153** | **chemo-response** | **16273092** |
| **Dressman et al.** | **HGU133A** | **117** | **OS, chemo-response** | **17290060** |
| **Patch et al.** | **Illumina HiSeq2000** | **80** | **OS, chemo-response** | **26017449** |
| **GSE15622** | **HGU133A 2.0** | **35** | **chemo-response** | **18068629** |
| **GSE28739** | **GPL7264** | **50** | **chemo-response** | **21799864** |
| **GSE66667** | **HGU133 Plus 2.0** | **36** | **chemo-response** | **24398046** |
| **GSE71340** | **Illumina HiSeq2500** | **11** | **chemo-response** | **27306793** |
| **GSE51373** | **HGU133 Plus 2.0** | **28** | **chemo-response** | **24237932** |
| **GSE23554** | **HGU133A** | **28** | **chemo-response** | **21849418** |
| **GSE114206** | **GPL13497** | **12** | **chemo-response** | **30056367** |
| **GSE9891** | **HGU133 Plus 2.0** | **260** | **OS** | **18698038** |
| **GSE32062.GPL6480** | **GPL6480** | **260** | **OS** | **22241791** |
| **GSE26712** | **HGU133A** | **185** | **OS** | **18593951** |
| **GSE53963** | **GPL6480** | **174** | **OS** | **25269487** |
| **E.MTAB.386** | **Illumina HumanRef-8 V2** | **129** | **OS** | **22348002** |
| **GSE17260** | **GPL6480** | **110** | **OS** | **20300634** |
| **GSE26193** | **HGU133 Plus 2.0** | **107** | **OS** | **22101765** |
| **GSE63885** | **HGU133 Plus 2.0** | **101** | **OS** | **24478986** |
| **GSE14764** | **HGU133A** | **80** | **OS** | **19294737** |
| **GSE30161** | **HGU133 Plus 2.0** | **58** | **OS** | **22348014** |
| **GSE31245** | **HGU95Av2** | **57** | **OS** | **16204010** |
| **GSE18520** | **HGU133 Plus 2.0** | **53** | **OS** | **19962670** |
| **GSE19829.GPL8300** | **HGU95Av2** | **42** | **OS** | **20547991** |
| **GSE32063** | **GPL6480** | **40** | **OS** | **22241791** |
| **GSE19829.GPL570** | **HGU133 Plus 2.0** | **28** | **OS** | **20547991** |
| **GSE154600** | **Illumina HiSeq 2500** | **5** | **chemo-response** | **32747365** |
